# A Summary Case Report on the Health Impacts and Response to the Pakistan Floods of 2010

**DOI:** 10.1371/currents.dis.cc7bd532ce252c1b740c39a2a827993f

**Published:** 2013-04-11

**Authors:** Omar Shabir

**Affiliations:** Barts and The London School of Medicine and Dentistry, Queen Mary, University of London, London, United Kindgom

## Abstract

In July 2010, Pakistan suffered nationwide floods after unprecedented monsoon rains overwhelmed the Indus basin. The ensuing floods claimed 1985 lives, injured 2946 people and affected over 20.2 million people. Seventy-eight out of 121 districts were affected and at one stage one-fifth of the country’s land was inundated with water. Indiscriminate damage was caused to housing, educational and health facilities, communication networks, power plants and grids, irrigation channels, agricultural land and livestock. Over 37 million medical consultations were reported within one year of the floods with acute respiratory infection, skin diseases, acute diarrhoea and suspected malaria forming the most common presentations. Rescue and relief operations were organised through the National Disaster Management Authority and a UN Cluster Approach was adopted for providing humanitarian assistance. The Office for the Coordination of Humanitarian Affairs (OCHA) played a pivotal role in coordinating relief efforts between cluster groups and providing communication platforms for identifying gaps and sharing information. This paper attempts to collate information available in the public domain into a summary report based on key principles described by Kulling et al. (2010) on health crisis reporting.

## 1. Introduction

The 2010 floods in Pakistan began as a result of unprecedented monsoon rains overwhelming the Indus basin. The ensuing floodwaters affected 78 out of 121 districts nationwide engulfing an area of 100,000 km^2^. The floods claimed 1985 lives, affected 20.2 million people, damaged 2.4 million hectares of agricultural land and damaged or destroyed 2.1 million houses and 515 health facilities.^1^ Indiscrimate damage was caused to state infrastructure including: transport and communication networks, water and irrigation channels, power and energy plants and grids. Over 37 million medical consultations were reported by the Disease Early Warning System (DEWS) within one year following the floods with acute respiratory infection (23%), skin diseases (11%), acute diarrhoea (9%) and suspected malaria (6%) forming the most common presentations in flood affected districts.^2^

ReliefWeb, an online database administered by the United Nations Office for the Coordination of Humanitarian Affairs (OCHA), provided humanitarian information to organisations and interventionists during the floods. The database was continuously updated with situational reports and bulletins by various stakeholders (e.g. UN agencies, NGOs) and enabled the humanitarian community to assess need and mobilise response to the disaster accordingly. However, much of the information published on the ReliefWeb database was fragmented and the number of summary reports documenting the floods in a holistic manner was limited. Furthermore many of these reports exhibited an inconsistency in format and content that did not follow a standardised method for reporting. Therefore their expediency to humanitarian organisation and interventions - although advantageous - can be probed.

Kulling et al. (2010) have proposed guidelines promoting a common structure and method for reporting health crises and critical health events. These guidelines require reports to include assessments of the status before the event, a description of the disaster and the subsequent damage, the relief and recovery responses to the events and identification of lessons that can inform preparations and interventions in future crises. Therefore a concise report on the Pakistan floods of 2010 based on key principles described by these guidelines can serve as a useful resource in analysing summary findings that may help to improve preparedness, planning and response for future crises whilst advancing international collaboration and learning.^3^

## 2. Methodology

This summary report is based on the key principles of health crisis reporting described by Kulling et al.^3^ The main focus of this paper is to provide a summary focus on the pre-flood status of Pakistan, the impact of the floods and the subsequent response efforts by the humanitarian community. All the data included in this report are available in the public domain. As academic literature on recent floods in Pakistan have proved to be scarce and fragmented, a literature search was conducted using multiple search engines and databases to enable a wider inclusion, and therefore consideration, of primary data for this report. Search terms synonymous with “Pakistan floods”, “Pakistan floods 2010”, and “Pakistan floodwaters” were employed. An initial literature search was conducted on the PubMed/Medline databases and all resulting and related papers and references were extracted and analysed. Reliefweb, a database of humanitarian information maintained by the United Nations Office for the Coordination of Humanitarian Affairs, was used to collect all information related to Pakistan floods of 2010. Bibliographies of reports and articles were followed and extracted. Information and literature available on stakeholder websites including UN agencies, governmental departments, non-governmental organisations and academic institutions were studied. A wider search of grey literature was carried out on the internet.

Inclusion and Exclusion criteria: All information from frontline sources involved in the Pakistan floods including UN organisation, governmental departments, non-governmental organisation and academic institutions were considered. However, newspaper and journalistic report articles were excluded.

This report forms part of a research project that I undertook as part my medical studies at Barts and The London School, University of London. I received supervision from Dr Paul Wilkinson of London School of Hygiene and Tropical Medicine and Professor Virginia Murray of Extreme Events and Health Protection at Health Protection Agency UK. This report was not funded or allied with any governmental or non-governmental organisation.

## 3. Pre-floods

**3.1. Country Profile**

Pakistan covers an area of 796,096 km^2^ and stretches from the Himalayan Mountains in the north to the Arabian Sea to the south. The country shares borders with Afghanistan and Iran in the west, China in the north-east and India in the east; and is made up of four large provinces: Balochistan, Khyber Pakhtunkhwa, Punjab and Sindh; and also a small federal capital territory and a group of federally administered tribal areas. Pakistan has a total population of over 178 million people.^4^ The Gross Domestic Product (GDP) of Pakistan is US$ 841 per capita with government expenditure on health of US$ 7 per capita forming a total expenditure on health of 2.9% of GDP.^5^

**3.2. Climate**

Pakistan’s climate is predominantly semi-arid to arid and is typically characterised by hot summers and cool or cold winters. The northern mountainous and hilly regions of the country are cooler than the warmer and topographically flat regions of the south. Pakistan, depending on geographical location, weathers four seasons: a cool and dry winter period (December to February), hot and dry spring (March to May), rainy summer or monsoon period (June to September) and a retreating monsoon period (October and November).^6^ The annual rainfall in the northern parts of Pakistan receives less than 250 mm per year as compared to 125mm in the south. Rainfall during the monsoon rains, which accounts for 59% of annual fall, can increase to 750mm in the plains and 625mm in the highlands.^7^^,^ 8

**3.3. The Indus River**

The Indus River originates in Tibet and travels westwards through India and Kashmir before entering Pakistan through the northern mountains. It then runs southwards through the centre of the country before emptying into the Arabian Sea. The basin stretches approximately 3,000 km in Pakistan and covers an area of 977,000 km^2^ (approximately 25% of total land mass). The river sources its water from annual rainfall, glacier melt from the northern mountains and a number of large tributaries including Shigar, Shyok, Gilgitm and Kabul rivers from the northern province and the larger Beas, Chenab, Ravi, Jhelum and Sutlej rivers from the Punjab province (Figure. 1).^9^

**Map showing the major rivers of Pakistan.10:**
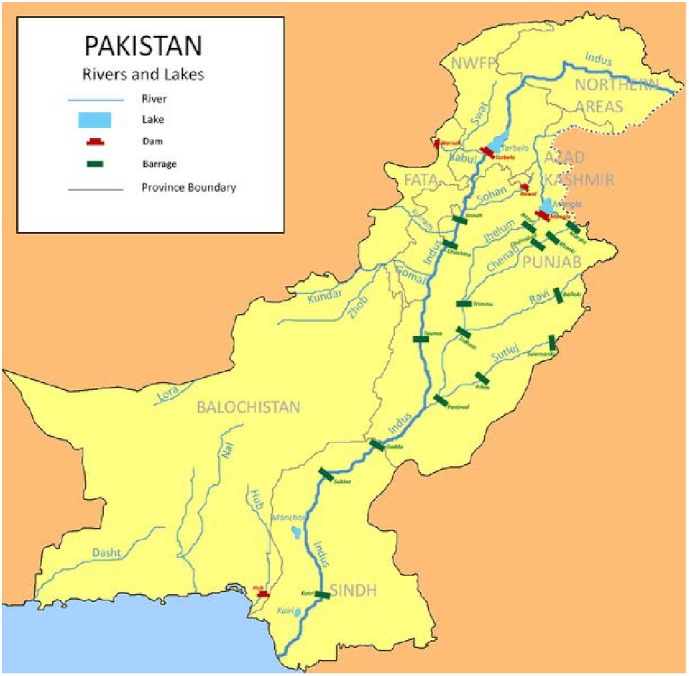

**3.4. Preparedness**

**3.4.1. Hyogo Framework for Action (2005-2015)**

The Hyogo Framework for Action (HFA) is a 10-year disaster risk reduction plan that has been adopted by 168 member states of the UN. It was established in 2005 and describes strategies aimed to reduce losses caused by disasters (e.g. lives, social, economic and environmental) by 2015. The guidelines list key activities to build national resilience and outline five priorities for action (PFA):

1. *“Ensure that disaster risk reduction is a national and a local priority with a strong institutional basis for implementation.”*
2. *“Identify, assess and monitor disaster risks and enhance early warning.”*
3. *“Use knowledge, innovation and education to build a culture of safety and resilience at all levels.”*
4. *“Reduce the underlying risk factors.”*
5. *“Strengthen disaster preparedness for effective response at all levels.” 11***

Pakistan became a signatory of the framework after the 2005 earthquake exposed vulnerabilities of the existing disaster risk management strategies adopted by the country. In 2006, the National Disaster Management Ordinance (NDMO) 2006 was introduced by the Government of Pakistan to provide a legal framework for disaster risk reduction at a federal, provincial and district level and included nine priority areas from the HFA. In 2010 the framework was enacted under the National Disaster Management Act 2010.^12^^,^ 13 Although institutional commitments to the PFAs have been attained at a Federal and Provincial level, the degree of achievement have varied for each strategy and action.^12^

**3.4.2. National Disaster Management Authority (NDMA)**

The NDMA, under the National Disaster Management Commission (NDMC), is responsible for devising guidelines and implementing programmes on disaster risk reduction, preparedness, capacity building, response and recovery. During disasters the NDMA acts a central hub for implementing, coordinating and monitoring disaster management. They communicate with all stakeholders to facilitate a collaborative response to the disaster (including Pakistan Disaster Management Authorities, Army, Governmental Ministries and Departments, NGOs).^14^

Some of the key functions of the NDMA include:

- “act as the implementing, coordinating and monitoring body for disaster management; **
- prepare the National Plan to be approved by the NDMC; **
- Implement, coordinate, and monitor implementation of the National policy; **
- lay down guidelines for preparing disaster management plans by different Ministries or department and the Provincial Authorities; **
- provide necessary technical assistance to the Provincial Governments and the Provincial Authorities for preparing their disaster management plans in accordance with the guidelines laid down by the national Commission;**
- coordinate response in the event of any threatening disaster situation or disaster;**
- Lay down guidelines for or give directions to the concerned ministries or Provincial Governments regarding measures to be taken by them in response to any threatening disaster situation or disaster;**
- Promote general education and awareness in relation to disaster management.” 13 **

**3.4.3. Provincial disaster management authroity (PDMA)**

Each province has a disaster management authority in the form of PDMA that operates under a Provincial Disaster Management Commission (PDMC).

Some of the key functions of the PDMA include:

- “formulate the provincial disaster management policy approved by the provincial commission;
- Coordinate and monitor the implementation of the National Policy, National and Provincial Plan;
- examine the vulnerability of different parts of the Province to different disasters and specify prevention or mitigation measures;
- lay down guidelines to be followed for preparation of disaster management plans by the Provincial departments and District Authorities;
- evaluate preparedness at all governmental and non-governmental levels to respond to disaster and to enhance preparedness;
- coordinate response in the event of disaster;
- give direction to any provincial department or authority regarding actions to be taken in response to disaster;
- promote general education, awareness and community training;
- provide necessary technical assistance or advise to District Authorities and local Authorities for carrying out their function effectively;
- advise Provincial Government regarding all financial matters in relation to disaster management;
- ensure that communication systems are in order and carry out disaster management drills regularly.” 13

**3.4.4. District Disaster Management Authority (DDMA)**

The DDMA, under the District Disaster Management Commission, is involved in executing disaster risk reduction programmes at a district and tehsil level (second-lowest tier of local government).

Some of the key functions of the DDMA include:

- “prepare disaster management plan including district response plan for the district;
- coordinate and monitoring the implementation of the National Policy, Provincial Policy, National Plan, Provincial Plan and District Plan;
- ensure that areas in the district vulnerable to disasters are identified and measures for the prevention of disasters and the mitigation of its effects are undertaken by the department of the Government at the district level and by local authorities;
- ensure that the guidelines for prevention, mitigation preparedness and response measures as laid down by the NDMA and the PDMA are followed by all district level Governmental departments and local authorities;
- setup, maintain, review and upgrade the mechanisms for early warning and dissemination of proper information to public;
- coordinate with local authorities local authorities to ensure that pre-disaster and post-disaster management activities in the district are carried out promptly and effectively;
- identify building and places which could, in the event of disaster situation be used as relief centres or camps and make arrangement for water supply and sanitation in such building or places;
- establish stockpiles of relief and rescue material or ensure preparedness o make such material available at a short notice.
- control and restrict movement of any person in a vulnerable or affected area;
- provide shelter, food, drinking water and essential provisions, healthcare and services;
- establish emergency communication systems in the affected areas;
- remove debris conduct search and carry out rescue operations;
- make arrangements for the disposal of unclaimed dead bodies.” 13

**3.4.5. Federal Flood Commission (FFC)**

The Federal Flood Commission (FFC) was created in 1977 in response to the severe floods of 1973 and 1976; which exposed vulnerabilities in existing disaster risk management and signified the importance for a national policy on the flood problem. In 1978, the FFC prepared the National Flood Protection Plan (NFPP) that set out to reduce flood losses, prioritise flood protection for areas of greatest economic risk, provide protection for areas outside the flood plains and improve existing flood protection facilities.^15^ Under the FFC, three 10-year plans have been implemented: NFPP-1 (1978-1988) mainly emphasised for the installation of structural flood protection measures (e.g. precipitation measuring systems, radars and improvements to telemetry networks); NFPP-2 (1978-88) mainly focused on the installation of more structural flood protection measures and the establishment of the National Flood Forecasting Bureau, now Flood Forecasting Division (FFD); and NFPP-3 (1998-2008) focused on strengthening of NFPP-2. NFPP-IV (2008- 2018) is currently under approval by the Planning Commission.^14^

**3.4.6. Flood forecasting Division (FFD)**

The Flood Forecasting Division (FFD) of the Metrological Department is the sole flood forecasting agency in Pakistan and is responsible for issuing forewarnings to relevant stakeholders (e.g. FFC, Provincial and National Disaster Management Authorities, Ministry of Water & Power, Combatant Generals' Headquarters (GHQ), Ministry of Defence) to prevent and mitigate damage to lives, property and infrastructure caused by floods. The FFD receive and process hydro-meteorological data from various national sources to prepare flood forecasts and warnings. The Water and Power Development Authority (WAPDA) and the Irrigation Department provide the FFD with rainfall and river flow data in the catchment areas of the river Indus and Jhelum and hydrometric flood data from the Tarbela, Chashma and Mangla dams.^14^ The data is processed every six hours and analysed to produce flood forecasts and reports.^16^

**3.4.7. Pakistan Army**

The Pakistan Army’s Corps of Engineers have the responsibility of providing assistance to civil authorities by operating rescue and relief operations during national disasters. The Pakistan Army is involved in all phases of flood mitigation from pre-, during- and post-floods. During the pre-flood preparatory phase, the Commander Corps of Engineers make regular inspections of flood protection structures. During floods the Corps Engineers are stationed at the FFD to monitor the flood situation and provide regular situational updates, forecasts and warnings to the designated Director General (DG) and all other CC Corps of Engineers. Units of the Army are deployed to target areas to carry out rescue and relief operations. It is the responsibility of the PDMAs to provide the army equipment during disasters (e.g. boats, life-jackets, tents, vehicles). Further meetings are held post-floods to assess performance and identify lessons for the future.^14^

**3.4.8. UN Clusters Approach**

In 2005, the UN’s Inter-Agency Standing Committee (IASC) established a ‘Cluster Approach’ to humanitarian assistance with the aim of improving predictability, timeliness and effectiveness of response and recovery. The initial nine clusters (Camp Coordination and Management, Early Recovery, Emergency Shelter, Emergency Telecommunications, Health, Hygiene, Logistics, Nutrition, Protection and Water Sanitation) with two added later (Education and Agriculture) consisted of groupings of UN agencies, non-governmental organisations (NGOs) and other international organisations and stakeholders around sectors and services that worked together to provide assistance during a humanitarian crisis.^17^

The cluster approach was first implemented, in its elementary form, in the aftermath of the 2005 earthquake in Kashmir. Each cluster was designated a lead agency and made responsible for coordinating deliverance of humanitarian assistance within that sector. These clusters held intra-agency forums to share information; formulate joint strategic plans and partnerships; define roles , responsibilities and delicate activities in order to avoid gaps in coverage, duplication of aid and delay in assistance.^18^ As a result of implementing the cluster system much experience had been gained and capacity formed in the northern regions of Pakistan.

**3.4.9. Disease Early Warning System (DEWS)**

The Disease Early Warning System (DEWS) was setup in Pakistan by the World Health Organisation (WHO) in collaboration with the Ministry of Health after the Kashmir earthquake in 2005. Its goals include reducing mortality and morbidity through early detection and response to alerts and diseases outbreaks (e.g. acute watery diarrhoea, cholera, malaria). DEWS receives surveillance data from 490 fixed and 554 mobile outreach centres and has been instrumental in predicting and controlling epidemics in Pakistan through weekly reporting of disease trends and responding to health alerts within 24 hours.^19^

**3.5. Hazard: Floods**

Pakistan is prone to natural disasters including floods, earthquakes, landslides and tropical cyclones. Riverine floods are a common phenomenon in Pakistan and are predominantly caused by concentrated rainfall in river catchments areas during the monsoon season; that are sometimes compounded by increased glacier melt. Monsoon currents from the Bay of Bengal in India and the subsequent depressions can result in heavy rainfall in the Himalayan regions of northern Pakistan. Furthermore the weather systems originating from the Arabian and Mediterranean seas can compound the heavy downpours in the north and inundate the Indus River and its tributaries.^20^

The Indus River has been responsible for 11 of the major floods in Pakistan including the floods of 1950, 1955, 1956, 1973, 1976, 1978, 1988, 1992, 1995, 1997 and 2005 (Figure 2).^15^^,^ 21 The floods of 1973 claimed 474 lives and destroyed over 3 million houses. The floods of 1976 caused 425 deaths and destroyed 10 million houses with a total cost to the economy of 6 billion rupees.^21^ The floods of 2005 that were caused by the warm weather and snowmelt in the northern regions killed more than 30 people and affected over 460,000 people nationwide.^22^

**Major floods of Indus River in Pakistan.15:**
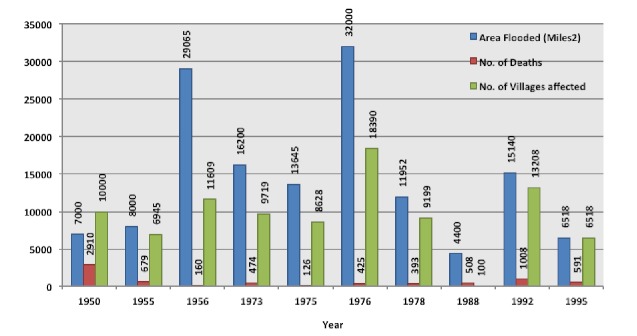

**3.6. Vulnerability**

**3.6.1. Housing**

The poor quality of housing stock in the rural areas of Pakistan makes them vulnerable to natural disasters. Houses in the northern mountainous regions are typically of kutcha (non-permanent) stock and constructed from mud or stone rubble making them feeble and unable to withstand extreme natural events. Even the pucca (permanent) are not exempt from these dangers due to poor structural design comprising heavy concrete slabs supported by thin walls constructed of sand and cement mortar.^23^ Furthermore, the majority of houses lining the Indus River in Punjab and Sindh along the heart of the country are made of basic adobe materials and inhabited by poor agrarian people. Only 24% of rural areas have access to piped water and the major sources for drinking water are tube wells, hand pumps and boreholes. Furthermore, 43% of rural households do not have any toilet facilities.^24^

**3.6.2. Environment**

Extensive deforestation, particularly in the northern mountainous regions around the Indus River, has resulted in soil erosion. The resulting reduction in vegetation has led to an increase in surface water run-off during monsoon seasons. Augmented by increased glacial melt, deforestation has resulted in the Indus River receiving a greater amount of water upstream. 25

**3.6.3. Flash Floodings**

Flash floods, as opposed to slow rising riverine floods, can cause considerable damage to lives, communities and property as they can present suddenly and without warnings. Flash floods in Pakistan have predominantly occurred in the mountainous ranges of the Himalayans, Kashmir and Gilgit-Baltistan due to steeply uneven topography and unpredictable climate; and in D.I. Khan, D.G. Khan and Kirther Ranges of Balochistan and Sindh Provinces as result of hill torrents and around the coastal areas of Sindh and Balochistan due to tropical cyclones.^20^

**3.6.4. Communicable Disease**

The most prevalent communicable diseases in Pakistan include acute respiratory infection, diarrhoea, polio, tuberculosis, hepatitis B and C, measles and vector-borne disease including malaria, Leishmaniasis and haemorrhagic fever (CCHF). The prevalence of malaria is greater in the rural areas and risk reduction behaviour is low with only 6% of households owning mosquito nets. Pakistan has an endemic problem with tuberculosis (297,000 cases reported in 2008) and polio.^24^

**3.6.5. Maternal and Child Health**

Pakistan has a high maternal mortality rate of 276 deaths per 100,000 women. Major causes of maternal death include haemorrhages and sepsis. Approximately 62% of all deliveries take place within the home and only 39% of deliveries have a skilled practitioner present. Child mortality rate (under 5 years old) is 94 per 1000 live births; infant mortality rate (under 1 year old) is 78 per 1000 live births; and neonatal mortality rate (under 28 days old) is 54 per 1000 live births. The main causes of infant death include pneumonia, sepsis, diarrhoea and meningitis.^24^ Immunisation rate in Pakistan is remarkably low with coverage of only 46%.^26^

## 4. During floods

**4.1. timeline**

On 20^th^ July 2010, the FFD issued the first of a series of flood warnings to low lying areas and districts of Punjab as result of widespread thunderstorms and heavy downpours generated by the annual monsoon season. The FFD advised all concerned authorities to take precautionary measures to protect human lives, infrastructure and property. Similar warnings were issued on almost a daily basis; however, the amount of time given between the issuance of the warning and the expected time for flooding varied and ranged from zero to several hours in the northern regions (KPK and Punjab) and a maximum of around 2 days for the southern regions (Appendix 1).

On 22^nd^ July, the first of the heavy monsoon rains hit the north-eastern regions of Balochistan causing flash floods and affecting 50,000 people. A second spell of heavy rains, which lasted for two months, followed on 27^th^ July and resulted in the FFD issuing high flood level warnings along the River Chenab in Punjab and River Kabul in Khyber Pakhtonkhwa. On 28^th^ July the FFD issued further warnings of heavy flooding in the Kalat, Sibi, Naseerabad and Zhob districts of Balochistan and DG Khan and Rajanpur districts and river Tarbella in Punjab and northern Khyber Pakhtonkhwa provinces.

On 29^th^ July, the Swat and Kabul rivers burst their banks resulting in the flooding of Nowshera and Charsadda districts and parts of Peshawar in Khyber Pakhtonkhwa. Furthermore, on-going rains in the northern regions caused landslides and further flash flooding in Khyber Pakhtonkhwa, Gilgit Baltistan and Pakistan-administered Kashmir causing loss of life and significant damage to property and infrastructure. By the morning of 31^st^ July the upper regions of River Indus around the Chashma district of Punjab had been flooded. As the floodwaters headed southwards, floods continued to devastate low-lying district surrounding the Indus basin (i.e. Bhakkar, Layyah, Muzaffargarh, DG Khan and Rajanpur). The total rainfall within the month of July was exceptionally high for the annual monsoon season with recordings of: up to 257mm in districts of Punjab; up to 280mm in Khyber Pakhtonkhwa; up to 58mm in Balochistan and up to 189mm in Gilgit Baltistan & Azad Kashmir.^27^

By 5^th^ August, as red alerted by the FFD two days earlier, the floodwaters reached the Sindh Province and drowned the district of Guddu. The FFD issued further red alerts of exceptionally high flooding in Sindh to the districts of Khairpur, Jacobabad, Sadiqabad, Shikarpur, Ghotki of Dadu Sukkur, Larkana, Nawabshah, Hyderabad and Naushehroferoze. By 7^th^ August, the floods had affected 15 million people nationwide.

On 9^th^ August, the Indus River overflowed its banks in Sindh causing wide scale destruction. By 26^th^ August, more river breeches had occurred in the district of Thatta and further flood warnings were issued for the district of Kotri. By 30^th^ August, more villages were submerged under water and approximately 1 million people fled from the floods in Sindh. On 13^th^ September, the large Manchar Lake breached its bank and overflowed causing serious flooding of the Jamshoro district (Figure 3).

**Maps showing the temporal progression of floodwaters from 30th July to 13th September 2010.28:**
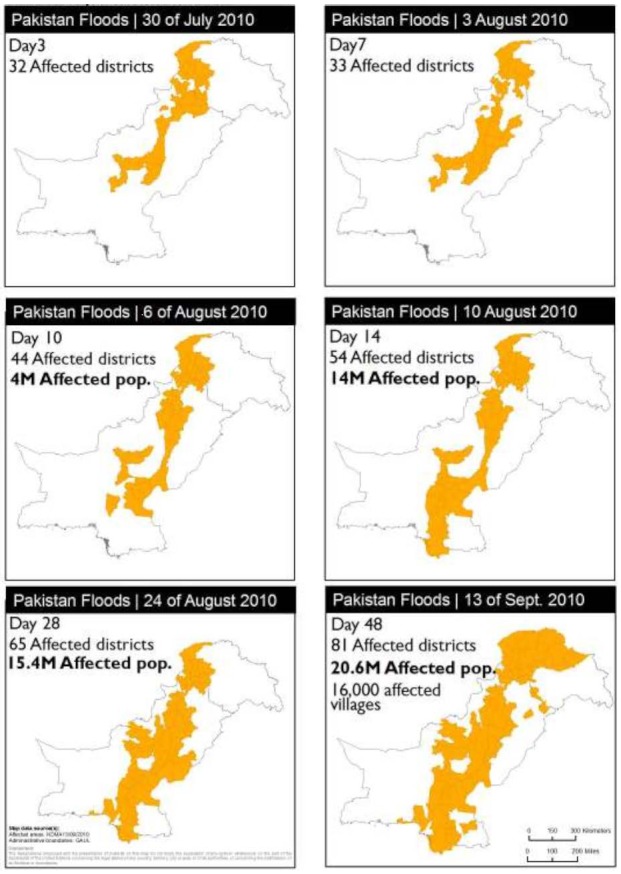

**4.2. Receding floodwaters**

The impact of the floods varied amongst the provinces depending on the nature of the floods (i.e. flash flood versus slow-rising riverine floods), the topography of each affected area and the different levels of preparedness measures. Where Khyber Pakhtunkhwa was only affected by flash floods, the provinces of Punjab and Balochistan were exposed to both flash and riverine floods and the Sindh province to mainly slow-rising riverine floods. Furthermore, the receding of floodwaters also varied depending on the topography of the region. In the topographically hilly Khyber Pakhtunkhwa and Balochistan floodwaters started to recede within days of their onset, however it took several weeks until mid-September in Punjab and several months in some areas of the flat plains of Sindh for the waters to completely clear.^29^

**4.3. Damage**

The Pakistan floods of 2010 have caused unprecedented damage to livelihoods, property, infrastructure and economy in the history of the country. The floods claimed 1985 lives, injured 2946 people and affected over 20.2 million people nationwide. Around 11 million people were displaced nationwide; with 7 million people from Sindh alone.^30^ Both flash and slow-rising riverine floodwaters affected 78 of the total 121 districts and at one point one-fifth of the country was submerged under water (Figure 4).^1^ Unparalleled damage was caused to housing stock, educational and health facilities, communication networks, power plants and grids, irrigation channels, agricultural land and livestock. The World Health Organisation (WHO) identified 23 of the most severely affected districts (Table 1).

**The number of affected districts in each province.1:**
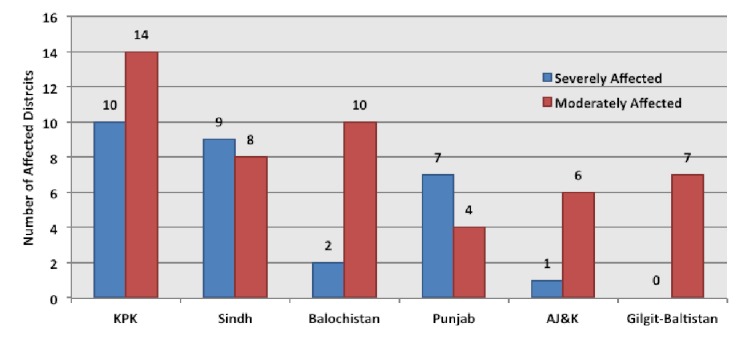

**Table 1: Worst flood-affected districts according to the World Health Organisation.24:** 

Province	Worst Affected District
**Sindh**	Jacobabad, Kashmore, Shikarpur, Gotki and Qambar Shahdad Kot ,Dadu, Jamshoro and Thatta
**Balochistan**	Jaffarabad, Nasirabad and Sibi
**KPK**	Nowshera, Charshada, Swat, Kohistan and Shangla
**Punjab**	DG Khan, Rahim Yar Khan, Layyah, Rajanpur and Bhakkar

**4.4. Impact on Human Health**

**4.4.1. Deaths and Injuries**

The floods caused 1985 deaths and injured 2946 people. Majority of deaths occurred in Khyber Pakhtunkhwa Province (1156; 58%), followed by Sindh (411; 20%), Punjab (110; 5.5%), Balochistan (183; 0.02%). The remainder of deaths occurred in the smaller tribal and administrated areas. Furthermore, the majority of deaths had occurred within the first 14 days with official data reporting 1271 deaths and 1334 injuries by 10^th^ August 2010.^31^ Sindh (1235; 41%) and Khyber Pakhtunkhwa (1198; 40%) had the most number of injured people (Figure 5).^29^

**Number of deaths and injuries caused by the floods.29:**
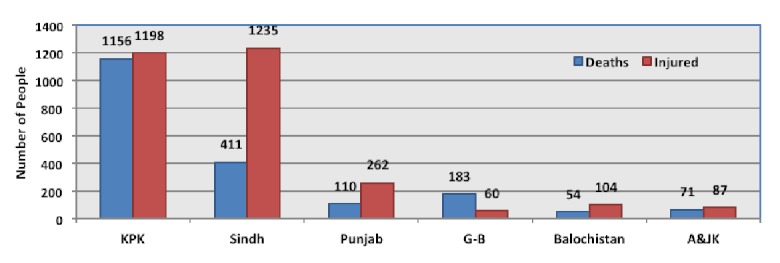

**4.4.2. Disease**

From 29^th^ July 2010 to 21^st^ July 2011 DEWS reported 37,391,802 medical consultations in flood affected districts. The most common illnesses included: acute respiratory infection (23%), skin diseases (11%), acute diarrhoea (9%) and suspected malaria (6%) (Figure 6).^2^ Other diseases reported by DEWS have included Tetanus, Meningitis, Leishmaniasis, Diphtheria, Acute Flaccid Paralysis, and Viral Hemorrhagic Fever.^32^

**The number of leading causes of seeking medical consultations reported to DEWS from 29th July 2010 to 21st July 2011.2:**
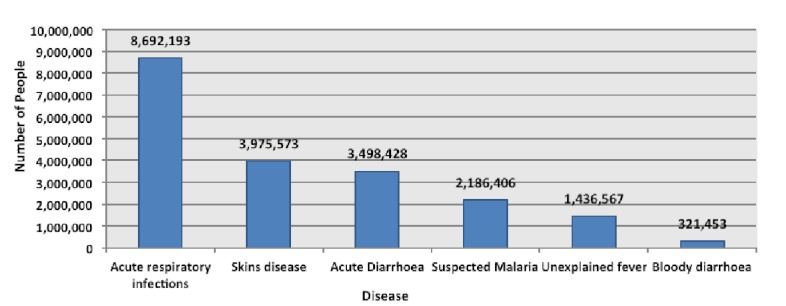

The incidence of acute respiratory infection increased within two weeks after the onset of the floods and reached a peak in early February 2011. The incidence of acute diarrhoea (AD) increased immediately after the floods and peaked towards the end of August 2010. After a falling trend from September 2010 to late February 2011 the incidence of AD began to climb again around mid-March 2011. The incidence of bloody diarrhoea (BD) remained generally low but constant with a slight increase between late August to mid-October. The number of suspected malaria cases increased between late-August 2010 till early January 2011 peaking in October 2010 (Figure 7).

**The proportional morbidity of priority diseases from flood-affected districts from 29th July 2010 to 2nd June 2011.33:**
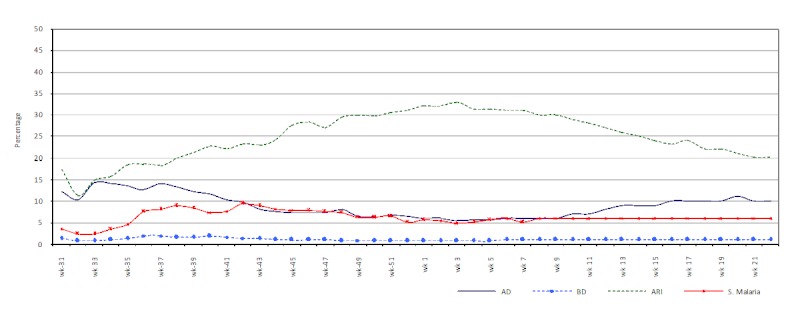

**4.4.3. Health Facilities**

The damage inflicted on the country’s public health infrastructure was considered to be mild to moderate with basic health units and medical dispensaries mostly affected. From a total of 9721 health facilities in Pakistan, 2957 were situated in flood-affected districts of which 515 were either damaged or destroyed by the floods.^34^^,^ 26 Khyber Pakhtunkhwa and Sindh suffered most proportional damage with 10.9% and 11.7% of total health facilities damaged or destroyed; respectively. About 8.9% of total provincial health facilities in Fata, 6.3% in Azad & Jammu Kashmir, 2.1% in Balochistan and 2% in Punjab were affected by the floods (Figure 8). Most of these healthcare facilities were located in rural areas and provided basic health services to the local population. Although most of the secondary healthcare facilities were unaffected by the floods, however the disruption caused to primary healthcare providers led to secondary healthcare facilities becoming inundated with greater demand for services. Furthermore it is estimated that around 35,000 female health workers had been displaced during the floods causing further disruption to the health service.^28^ The total cost of damage to health facilities has been estimated around $50 million.^1^

**Number of health facilities partially or completely damaged by the floods in each province.26:**
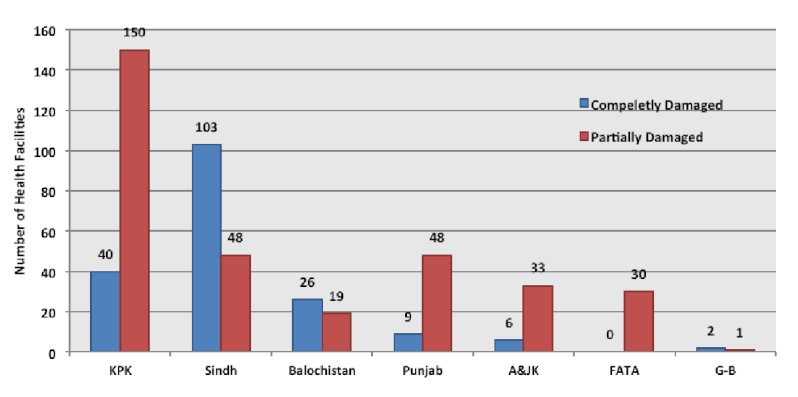

**4.4.4. Food and Nutrition**

The World Food Programme Flood Impact Assessment reported that at least 10.1 million people required emergency assistance with food of which about 3.6 million people would require long-term assistance. Risks of diarrhoeal outbreaks caused by water borne diseases, poor feeding and loss of livelihoods predisposed the displaced and affected population to acute and chronic malnutrition.

The pre-existing levels of malnutrition in the population (13.2% of Global Acute Malnutrition (GAM) and 3% of Severe Acute Malnutrition (SAM)) had been exacerbated by the floods. Provincial surveys showed that the level of GAM in northern Sindh had increased to 22.9% and 26% in southern Sindh. A total of 13.2 million people required nutritional attention following the floods. Furthermore, about 2.8 million (14%) children under the age of 5 years old and 1.6 million (8%) pregnant and lactating women formed part of the total affected population 35

**4.5. Impact on other Sectors**

The floods affected 78 out the 121 districts of Pakistan inundating over 160,000 km^2 ^of land mass causing unprecedented damage to housing, educational and health facilities, communication networks, power and energy plants and grids, irrigation channels, agricultural land and livestock (Figure 9). The total cost of damage was estimated to be around US$ 10 billion. Sindh suffered the most damage estimated to be around US$ 4.3 billion, followed by Punjab at US$ 2.5 billion, KPK at US$ 1.7 billion and Balochistan at US$ 620 million (Figure 10).^26^

**Estimated total cost of damage according to sector in US$ million.26:**
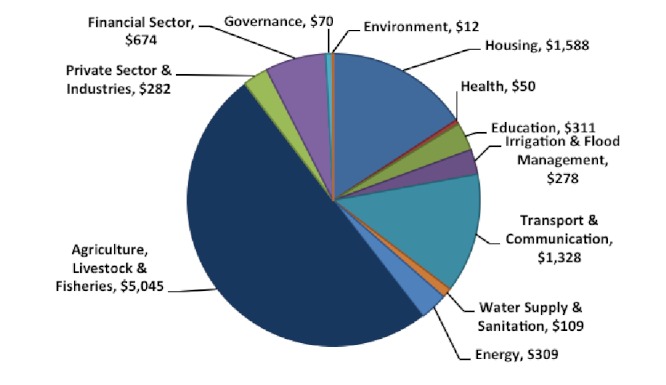

**Estimated total cost of damage according to Provinces in US$ million.26:**
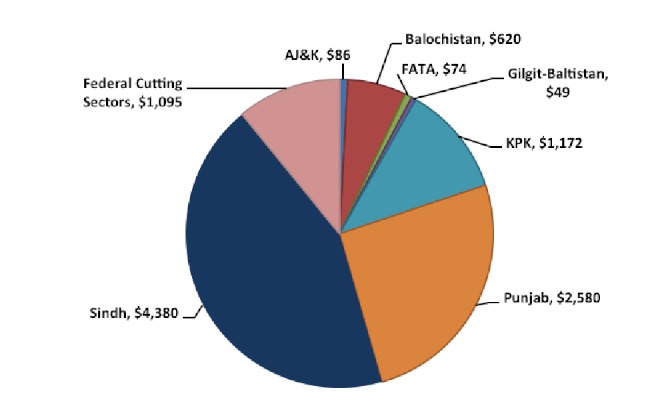

**4.5.1. Housing**

Over 1.6 million houses were either damaged or destroyed by the floods. Approximately 1.45 million of the affected houses were of *kutcha* stock and around 850,000 had been completely destroyed. Around 156,000 units of *pucca* houses were affected by the floods of which around 65,000 were completely destroyed. Sindh suffered the most losses to housing with over 800 thousand houses (24% of pre-flood stock) becoming damaged or completely destroyed. About 375,000 houses (9% of pre-flood stock) in Punjab, 250,000 houses (9% of pre-flood stock) in Khyber Pakhtunkhwa, 80,000 houses (14% of pre-flood stock) in Balochistan were either damaged or complete destroyed by the floods.^28^ The total cost of damage to housing has been estimated at around $1.158 billion (Figure 11).^26^

**Number of houses destroyed in each Province.26:**
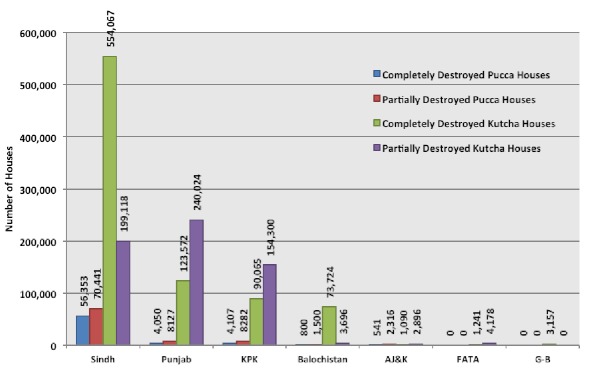

**4.5.2. Water Supply and Sanitation**

The severity of damage caused to water supply and sanitation infrastructure varied across the provinces. Flash floods in Khyber Pakhtunkhwa caused most structural damage to water supply networks and infrastructure including pump houses, store tanks and water pipes. Whereas the slow-rising riverine floods in Sindh caused more damage to electrical and mechanical components, pump houses and machinery. Sanitation infrastructure was most affected in the south with extensive damage inflicted on sewerage and drainage systems.^26^ As water and sanitation is a major determinant of communicable diseases, the impact of the damage on this sector had wide-reaching health implications. The total cost of damages in this sector is estimated to be around $109 million.^1^

**4.5.3. Irrigation**

Severe damage was caused to the country’s 50 years old irrigation system that included irrigation channels, canals, drains and public tube wells. The Sindh province suffered most damage costing around $136.9 million followed by Khyber Pakhtunkhwa at around $68 million.^1^ The overall damage to the irrigation is estimated to be around $278 million.^26^ The direct damage to the irrigation infrastructure compounded with water siltation and water-logging of agricultural lands have also caused great destruction to crops.^26^

**4.5.4. Education**

The floods caused indiscriminate damage to educational facilities that account for 6.2% of total institutions in Pakistan. A total of 10,407 educational institutions were severely affected by the floods of which 6,666 fully destroyed and 3,741 partially damaged. A total of 18.5% of total educational institutions were either damaged or destroyed in the Sindh province and a further 8.8% in Punjab, 12.9% in Balochistan and 5.6% Khyber Pakhtunkhwa.^26^ The total cost of damage to education sector has been estimated at around $311 million.^26^

**4.5.5. Transport and Communications**

The damage incurred to the transport and communication networks (including roads, bridges, railways, airports and telecommunication infrastructure) have had multiple implications ranging from mobility of the affected population to access to basic services (e.g. health, education, public services and markets). Approximately 25,000 km (10%) of road networks and 1,225 km (16%) of railways have been damaged by landslides and floods, incurring losses of $1.2 billion and $60 million, respectively. Damage to the telecommunications infrastructure, which includes cables, transmission towers and optic fibre networks is estimated around $35 million. The total damage incurred by this sector has been estimated to be $1.3 billion.^26^

**4.5.6. Agriculture, Livestock and Fisheries**

Agriculture, livestock and fisheries have been the most severely affected sector accounting for 44% of total damage inflicted by the floods. The damage caused to agriculture and livestock varied in each province according the nature of the flood. The unexpected flash floods in the mountainous areas of Balochistan, Khyber Pakhtunkhwa, Pakistan-administrated Kashmir and Gilgit-Baltistan overwhelmed the local population and resulted in the sweeping away of people, houses, livestock, feed stores and food. The slow-riverine floods in the topographically flat plains of Sindh and Punjab together with longer advance warnings by the FFD enabled the population to relocate and save most of their livestock. In total about 1.5 million animals and 10 million poultry birds were lost to the floods.^26^

Waterlogging and siltation of agricultural lands caused great destruction to crops. According to the Preliminary Damage and Needs Assessment report, damage to crops accounted for 89% (i.e. 2.1 million hectares of mostly cotton, rice, sugarcane and vegetables) and live stock 11% of the overall losses.^1^ The Sindh province suffered 46% of the total damage to the agriculture, livestock and fisheries sectors, followed by Punjab (36%), Khyber Pakhtunkhwa (8%) and Balochistan (8%). The total damage to this sector is estimated at $5 billion.^26^

**4.5.7. Environment**

Forests, wetlands and other natural systems have suffered damages of around $12 million.^26^ Furthermore, the floods had serious implications on environmental health though contamination of drinking water, accumulation of solid waste and proliferation of disease vectors around stagnant waters.^29^

**4.5.8. Energy**

Pakistan's electricity sector suffered major damages to hydroelectric power generation stations, distribution grid and networks. About 3.5 million people, predominately from Sindh and Khyber Pakhtunkhwa, were left without power. The total damage to the electricity sector was estimated to be around $155 million. Pakistan’s petroleum sector, although representing only 1% of the annual oil imports, also suffered damages totalling around $155 million. Over 240,000 people were cut off from gas supplies, and a number of power generation facilities were temporarily suspended following shortages of oil. Total damage to the energy sector is estimated around $309 million.^26^

**4.5.9. Private Sector and Industries**

Whilst the major industrial centres of the country were spared, the floods severely affected small and medium business enterprises. Shops, industrial factories and workhouses received the largest share of the damage estimated at around $282 million. Sindh and Punjab suffered most damage to commercial, agricultural and farming sectors (i.e. cotton, sugar, rice and flourmills). Losses of around 2 million bales of cotton had knock-on effects on the textile industry; which forms one third of the country’s manufacturing sector. Khyber Pakhtunkhwa suffered most damage to its marble, mining, silk, furniture manufacturing and tourism sectors.^29^ Furthermore damage to the private sector has had serious implications on the labour forces incomes and livelihoods and overall resilience. The total cost to this sector is estimated at around $282 million.^26^

## 5. Post-floods

The initial response to the floods was coordinated by the NDMA and carried out by PDMAs, DDMAs, local population, philanthropist and the army. Much experience and capacity had already been formed following the Pakistan earthquake of 2005 that allowed for early mobilisation of response; particularly in KPK where the recovery phase was still in progress following the earthquake. However, as the floods continued to overwhelm and exhaust the country’s resources and capacity, the Government of Pakistan sought help from the international community. By early August 2010 the UN appealed for $459 million through the Pakistan Initial Floods Emergency Response Plan (PIFERP) to cover the immediate relief period and at the heed of the Pakistan government prioritised four clusters (Food, Health, Shelter and Water, Sanitation and Hygiene (WASH)).^10^ Within weeks, the humanitarian response to the Pakistan floods became the largest relief operation launched by the international community in recent history comprising various United Nation agencies, international non-governmental organisations, foreign government and donors. However, due to initial poor flow of donations the initial response to the floods was heavily hampered, especially in the southern provinces. A revised PIFERP requested over $1.9 billion to cover the early recovery period lasting approximately one year.^36^ Eventually the UN increased the number of clusters to assist flood-affected people. The NDMA also arranged for visas-on-arrival for relief workers and exempted tax and duty for imported relief items.^1^

**5.1. Rescue and Relief phase**

The Rescue and Relief Phase started immediately after the onset of the floods in July 2010 and aimed to reduce mortality, morbidities, disease and malnutrition in the affected population.^14^ The NDMA, together with the assistance of the army, initiated a rescue operation that included moving stranded people to safety, transporting people that required medical attention and distributing emergency relief supplies (including tents, food, water, sleeping mats, hygiene kits and medical services) (Table 2). The army deployed 60,000 troops, utilised their entire fleet of C-130 planes in the initial operation. Marine support was provided through 1000 boats including 50 life-saving naval boats. The army was also involved in repairing transport infrastructure (e.g. roads, bridges) and setting up mobile medical camps.^37^ As a result 800,000 people were rescued within one month following the initiation of the Rescue and Relief Phase.^38^ By 17^th^ September 2010 a total of 1.4 million people were either rescued or evacuated from the floods.^1^

A total of 88 helicopters (including 48 from friendly countries), 4 hovercrafts and 1238 boats were deployed during the initial rescue operation. A total of 5928 relief camps were established nationwide that sheltered over 3 million people at the peak of the disaster. These had decreased to 103 relief camps and 91,773 people by 31^st^ December 2010 (Table 3).^1^

**Table 2. Details of NDMA mediated Relief Support up to 31st December 2010.1:** 

Achievements	Number
**Food**	
Food items	0.41 million tons
Ready to eat meals	10.76 million
**Shelter**	
Tents	489,177
Tarpaulin	931,317
Blankets	1,918,483
Plastic mats	129,537
Mosquito nets	290, 262
Kitchen sets	412,401
**Health**	
Medicines	428 tons
Hygiene kits	160,470
Water purification tablets	250 million
Medical consultations	20.11 million
**Water and Sanitation Hygiene (WASH)**	
Water purification kits/buckets	11,341
Water purification plants/units	3896
De-watering pumps	45

**Table 3. Number of people in relief camps from 27th July to 31st December 2010.1:** 

	No. of Relief Camps		No. of People in Relief Camps		% of Returning People by 31/12/10
Province	During Peak	On 31/12/10	During Peak	On 31/12/10	
Punjab	327	0	116,295	0	100%
Sindh	4,682	73	1,815,961	76,421	96%
KP	825	0	915,463	0	100%
Balochistan	67	30	150,343	15,352	90%
AJ&K	2	0	900	0	100%
**Total**	**5,928**	**103**	**3024,432**	**91,773**	**97%**

The relief efforts were further augmented with the formation of clusters that initially prioritised food, health, shelter and WASH but were later rolled out to include other sectors (Appendix 2). This paper will summarise the response of clusters directly related to human health: Health, Food, Nutritional, WASH and Shelter.

**5.1.1. Health Cluster**

The health cluster led by WHO and the Ministry of Health had the objective of providing emergency health assistance in the flood-affected areas of KPK, Balochistan, Punjab, Sindh, the small tribal areas and the state of AJK. During the initial Rescue and Relief phase a total of over 11.8 million medical consultations were reported from 29^th^ July to 30^th^ December 2010.^39^

Within one year from the onset of the floods over 37 million medical consultations were reported by DEWS, between July 29^th^ July 2010 to 21^st^ July 2011, from 73 out of 78 flood affected districts.^2^ The cluster provided essential medicine to over 14 million people and responded quickly to disease alerts, controlling over 480 outbreaks as identified by DEWS.^40^ The most common disease presentations included: acute respiratory infection, skin diseases, acute diarrhoea and suspected malaria. Over 10.3 million water disinfection chemical kits have been distributed.

**5.1.2. Shelter Cluster**

The movement of people from flood-affected districts led to the establishment of 5928 relief camps to shelter displaced communities. By 31^st^ December this number had reduced to 103 as about 97% of people returned homes with 91,773 people remaining in the camps.^1^ Up until March 2011 the Shelter Cluster have reportedly distributed 1.36 million plastic tarpaulins and 381,000 tents to over 1 million households (approximately 66% of the total estimated number of houses damaged or destroyed by the floods) and 14,520 one room shelters and 24,111 transitional shelters (i.e. lightweight structures that can be relocated) were set up. However, only 67% of the emergency shelter needs had been met. Punjab has received the most coverage with (93%) while the shelter cluster in the Sindh province has only reached 51% of the target amount.^41^

**5.1.3. Food Cluster**

About 10.1 million people were in need of food following the floods. By December 2011, the food cluster, headed by the World Food Programme (WFP), distributed over 350,000 metric tonnes (mt) of food.^42^ By February 2011, over 480,000 metric tonnes of food had been distributed in 65 districts in the form of monthly rations (in accordance with SPHERE standards for food security, nutrition and food aid).^35^ About 8.8 million people (including 4.3 million women and girls and 1.2 million children under the age of 5 years) had been assisted at least once and an average of 6 million people were reached on a regular basis.^35^ Food and cash for initiatives were also implemented to help rebuild agricultural infrastructure and rehabilitate communities.^43^

**5.1.4. Nutrition Cluster**

The Nutrition Cluster established 625 therapeutic centres that comprised of 597 outpatients therapeutic clinics and 28 stabilisation centres. By March 2011, the Nutrition Cluster had screened over 1.29 million children under the age of 5 years and over 492,000 pregnant and lactating women for malnutrition.^35^ By August 2011, the screening programme identified 95397 severely malnourished children, 256,226 moderately malnourished children and 159,750 pregnant or lactating women who were put onto the feeding programs.^40^

**5.1.5. WASH Cluster**

Approximately 13.1 million flood-affected individuals required the provisions or services of the WASH cluster. Key priorities included installing permanent WASH facilities (e.g. safe disposal of excreta), restoring and exceeding pre-flood WASH coverage in the affected districts and hygiene education to promote sustainability.^29^ The WASH cluster have provided drinking water to 2.5 million people, over 400,000 hygiene kits, 3 million bars of soap and have provided education, health and hygiene promotion to over 750,000 people.^29^

**5.2. Early Recovery Phase**

The Early Recovery Phase started in parallel to the initial relief phase and was included in the Pakistan Flood Relief and Early Recovery Plan in November.^28^ The NDMA, United Nations Development Programme (UNDP) and UN Humanitarian Coordinator formed the Early Recovery Working Group (ERWG) to aid recovery in 29 of the most affected districts. The ERWG, setup at federal, provincial and district level, consists of 8 Sectorial Working Groups (SWG) focused on 8 prioritised sectors (Agriculture & Food and Security, Health & Nutrition, Education, Water & Sanitation, Housing, Governance, Non-Farm Livelihood and Community Infrastructure) and 4 Thematic Groups based on 4 cross-cutting themes (Disaster Risk Reduction, Gender, Environment and Protection. The SWGs and TGs devised strategies for each sector and thematic group after conducting a Map and Gap Analysis to identify early recovery needs, challenges, response, funding and funding gaps.^44^ On 15^th^ April 2011, the Early Recovery Phase was formally initiated through the Strategic Early Recovery Plan that described sectorial and thematic strategies to address the gap between relief and long-term recovery and rehabilitation.

**5.2.1. Health and Nutrition**

The Health and Nutrition SWG focused on screening and providing nutritional support for moderate to severely malnourished children and pregnant and lactating women in 29 priority districts. The Health and Nutrition SWG identified 8 million people, including 1.4 million children under 5 years old and 1.4 million women who needing access to health care. Capacity was also developed through the restoration and rehabilitation of healthcare facilities. Temporary structures, medical supplies, and human resource support (e.g. female staff) was provided to manage with the increased number of patients.^45^ Further partnerships had been formed with UNICEF and World Food Programme to make up for the short fall in capacity.^29^

Up and until October 2011, 685 Outpatient Therapeutic Program (OTP) and Stabilization Centers (SC) had been established. Over 5.28 million children under the age of 5 years had been screened of which 127435,589 were included in OTP/SC and 4.3 million admitted in the Supplementary Feeding Program (SFP). Over 1.6 million pregnant lactating women had been screened for malnutrition of which 2.52 million were admitted in the Supplementary Feeding Program (Table 4).

**Table 4. Cumulative progress of the Health and Nutrition SWG.46:** 

**Province**	**No. of Sites (OTP & SC)**	**No. of Children Screened**	**Total No. of Children in OTP/SC**	**No. of SFP Site**	**No. of SFP Admissions Children**	**No. of PLWs Screened**	**No. of PLW’s Admitted in SFP**
**Balochistan**	0	63726	7696	0	21613	99722	25257
**KP**	29	1058805	12190	29	62806	372567	33093
**Punjab**	203	1841895	69685	202	223466	568594	119550
**Sindh**	453	2325459	37488	453	127704	573301	74780
**Total**	685	5289885	127059	685	435589	1614184	252680

**5.2.2. Water and Sanitation**

With the passing of the initial relief and recue phase, the emphasis shifted onto recovery and strengthening of institutional capacity to achieve long term health and development goals and supporting the return of displaced people.^29^ Sanitation has been the main focus of the Water and Sanitation SWG work. Their work has included repairing damaged water supply networks, installing or repairing hand-pumps and constructing latrines for the affected population. Much emphasis has also been placed on hygiene awareness through educational and training programmes. Until August 2011, 1.6 million homes were reached with hand pump installation or repair, 30,000 households have been provided with latrines and 1.2 million households have received educational sessions on hygiene.^40^

**5.3. Early recovery in Other sectors**

**5.3.1. Agriculture and Food Security**

The Agriculture and Food Security Sectorial Working Group led by the World Food Programme has been involved in ensuring food security for the affected population and building agricultural assets of local communities. From February 2011 to July 2011 food and cash for work initiatives were introduced to enable affected communities to rebuild agricultural infrastructure. About 100,300 households have benefitted from the cash for work initiative. About 768,680 households were provided with crop/vegetable packages and 327,340 households with livestock.^44^

**5.3.2. Housing**

On 31^st^ March 2011 the Shelter Cluster was handed over to the Housing Early Recovery Group (HERG) after being led by the International Organisation for Migration (IOM) during the Rescue and Relief Phase. The main strategy has been to focus exclusively on houses that were completely destroyed by building cost-effective and durable one-room shelters made from mud and brick per destroyed house. Up and until August 2011 453,293 one room shelter units have been constructed and around 63,700 transitional shelters have been completed.^40^ Emphasis has also been given on training programmes and centres that provide technical assistance to families building their own homes.^41^

**5.3.3. The Watan Card Scheme**

The Government of Pakistan established the Watan Card Scheme to enable the delivery of cash directly to the flood affected families. ATM cash cards were registered and distributed to affected families who were then able to obtain cash from ATM machines. Around 1.5 million households benefited from the scheme receiving sums of approximately £150 each. This scheme also allowed for money to be injected directly into the local economy.

**5.4. Pakistan floods 2011**

At the time of completing this report, the new onset of floods in the southern regions of Pakistan in 2011 had undoubtedly compromised the recovery and rehabilitation phase following the floods of 2010.^47^ The floods, triggered by heavy monsoon rains, have caused 486 deaths, injured 753 people and affected a total population of 5.15 million people in Sindh alone. Extensive damage has been caused to nearly 800 thousand houses, 200 health facilities and 2.28 million acres of agricultural lands.^48^ A comprehensive review of the impacts and effects of the floods 2011 in Pakistan and the post-flood relief and recovery efforts are beyond the scope of this paper.

## 6. Discussion

The floods of 2010 in Pakistan have caused unprecedented damage and affected over 20 million people, however the impact on health (i.e. mortality and morbidity rates) has been relatively low when compared to other natural disasters in recent history (e.g. Asian Tsunami in 2004 Hurricane Katrina in 2005). This could be attributed to three factors: the early warning system by FFD; the type of floods (flash floods versus slow-rising riverine floods); and the timing and dispatch of early response and recovery efforts to the disaster.

Flash floods that devastated the northern mountainous regions of KPK were responsible for more deaths (1,156) than the slow-rising riverine floods in the south. Furthermore, the number of deaths in Punjab (110) and Sindh Province (411) were remarkably low despite having a larger affected population. This discrepancy in mortality rates could be attributed to range of advance warning given by the FFD. The southern provinces benefitted from longer advance warnings compared to the northern regions. This supports the importance of an effective and timely advance flood warning system to allow people adequate time to prepare and relocate. The NDMA concedes that the current early warning system in Pakistan is of limited nature and can provide a forecast range of 3-4 days; however, coverage is almost non-existent in the north-western region and around the coastal belt of Balochistan.^42^ Further investment is needed in enhancing early warning systems through the application of modern technology for developing a more comprehensive system for monitoring and archiving data. More, infrastructural investment is needed in developing more efficient channels for disseminating warnings to vulnerable communities. The current system relies upon police wireless networks in police stations, the Forestry Department, mosque committees and other grass root organisations and the growing use of mobile (GSM) networks.^12^

The sudden and violent nature of flash floods, as seen in the northern mountainous region of KPK and Balochistan, have been responsible for more deaths - to both humans and livestock - than the slow-rising riverine floods that devastated Sindh and most of Punjab. The exact cause of death has been difficult to verify during the course of this report - as the information is not readily available - however review papers show that the leading cause of death by flash floods is drowning.^49^ Although the timing of flash floods is difficult to predict it is undeniable that they can be a major determinant of human health. Therefore a comprehensive risk analysis and hazard map should be drawn up so vulnerable communities can be identified and appropriate disaster risk reduction intervention can be implemented (e.g. informing, motivating and involving communities' people in all aspects of disaster risk reduction)

The swift and immediate responses by the NDMA and the military, especially in KPK, may have saved lives that may have been otherwise been lost. The capacity to respond to natural disasters had already been developed by the PDMAs of the northern regions following the 2005 earthquake in Kashmir. This gained knowledge and experience was reflected in the relief efforts during the July 2010 floods. It was felt by stakeholders (e.g. NGOs) that the deployment of relief response was quicker in the northern regions than in the southern provinces where sufficient warning time had been issued in advance of the floods. This shows that much more capacity building is required at a national and provincial level to ensure response is equal, measured and effective.

The United Nations led health cluster approach to coordinating humanitarian assistance to disaster victims has had mixed reactions from stakeholder groups. Although it is generally accepted that the approach had improved since the earthquake in 2005, it was commented that some clusters – which comprised over 600 agencies - lacked experienced leadership. This impacted on cluster meetings, which, rather than being utilised as a forum to coordinate assistance served as platform to share information. As a result of communication failure there were gaps in coverage, duplication of aid in certain areas and delays in assistance.^29^^,^ 37

An overwhelming case has been made for further investment in disaster preparedness and risk reduction. Pakistan needs to continue implementing strategies of the Hyogo Framework for Action (HFA). Although much progress has been achieved through institutional commitments, the Government of Pakistan needs to invest in emergency preparedness, response and recovery programmes.

## 7. Lessons Identified

The NDMA have been central to coordinating response to the floods of 2010. The NDMA initial rescue operation pays testament to the value of local capacity and leadership. However, where response coordinated by NDMA was swift in the northern regions of Pakistan, the response in Sindh was much slower despite adequate early warnings issued by the FFD. The NDMA need to invest in building capacity in Sindh and Punjab and disaster risk reduction measures in all provinces.

The Watan scheme was an innovative idea that helped to put cash directly in the hands of those in need. However, the scheme was fraught with bureaucracies and red tape that delayed the money reaching its intended beneficiaries. The registration system relied on presenting a national identity card on application. As majority of the people affected were poor and did not own an identity card they were excluded from being enrolled on to the scheme. The scheme could be enhanced by removing some of these barriers to make it more equitable. The government should carry out a registration process of all the people in the area as a preparedness measure for future floods.

The Clusters have achieved varying levels of success. There seems to have been a lack of clear leadership amongst the clusters, especially when some clusters have more than 600 humanitarian agencies grouped together. Initiatives that can rank agencies according to their leadership strengths and experiences should be explored and also structure the size of the meetings to allow for efficient communication and information sharing.

## 8. Conclusion

The Pakistan floods of 2010 affected over 20.2 million and claimed the lives of 1985 and injured 2946 people. The floods affected 78 out of 121 districts and at one stage submerged 20% of country total landmass underwater causing total damage of over $10 billion. KPK and Balochistan suffered predominantly from flash floods; whereas Punjab and Sindh suffered mainly from slow-rising riverine floods. The amount of fore warning disseminated to vulnerable communities was inconsistent throughout the country. The northern provinces of Pakistan were disadvantaged with shorter advance warnings than the southern provinces. However, response to the floods was much swifter in the northern regions as compared to the southern regions. The NDMA coordinated all response efforts and the UN Cluster approach was adopted to provide humanitarian assistance to affected communities. The initial Rescue and Relief Phase was discontinued on 31^st^ January 2011; except in 27 most affected districts where eight Sectorial Working Groups (SWG) were established in April 2011 to start the Early Recovery Phase. Unfortunately, recovery and rehabilitation efforts have been hampered by the recent floods of 2011 that have affected 5.2 million people and destroyed over 1 million houses in Sindh. The floods of 2010, and indeed 2011, have exposed vulnerabilities in the country disaster risk reduction system where further investment is required in emergency preparedness, response and recovery programme.

## Appendix 1

### Flood Forecasting and Warnings Time by the Pakistan Metrological Department. [1]

[1] Pakistan Metrological Depart. Government of Pakistan. Rainfall statement July 2010 Accessed on 21^st^ June 2011 via: http://www.pakmet.com.pk/FFD/index_files/rainfalljuly10.htm [online].

**Table:**

Date	Time	Hours notice	Forecast /Warning		Regions/Areas Affected
**20/07/10**	14:00		Widespread thunderstorm/rain at times heavy that may cause flash flooding in low lying areas		Lahore, Gujranwala, Sialkot, Rawalpindi, Peshawar, Kohat and Mardan (Kalpani Nullah) divisions of Punjab Province.
**22/07/10**	13:20	1:40	River Chenab expected to attain High Flood levels of at: Marala (15:00 hrs on 22/07/10 to 0300 hrs to 23/07/10), at Khanki (0100 hrs on 23/07/10 to 1400 hrs on 23/07/10) and at Qadirabad (0700 hrs of 23/07/10 to 20:00 hrs on 23/07/10		Riverbeds in Gujranwala, Sialkot, Gujrat, Hafizabad, Mandibahauddin districts of Punjab to be inundated.
**27/07/10**	15:30	12:00	River Chenab expected to attain High Flood levels of at: Khanki (03:00 hrs on 28/07/2010 to 15:00 hrs on 28/07/2010); Qadirabad (09:00 hrs on 28/07/2010 to 21:00 hrs on 28/07/2010)		Riverbeds in Gujranwala, Sialkot, Gujrat, Hafizabad, Mandibahauddin districts of Punjab to be inundated.
**27/07/10**	19:42	1:18	Moderate flooding likely in hill torrents (21:00 hrs on 27/07/2010 to 08:00 on 28/07/2010)		DG Khan and Rajanpur Districts of Punjab Province
**28/07/10**	08:30	00:30	Likely Heavy flooding in Hill Torrents (09:00 on 28/07/10 to 09:00 on 29/07/10)		Kalat, Naseerabad, Sibbi and Zhob division of Balochistan
**28/07/10**	08:30	00:30	Likely Moderate to Heavy flooding in Hill Torrents (09:00 hrs on 28/07/2010 to 09:00 hrs. on 29/07/2010)		DG Khan and Rajanpur Districts of Punjab.
**28/07/10**	14:50	02:10	Heavy rainfall expected in upper areas of River Jhelum. River Jhelum at Mangla is likely to attain High to Very High flood level (17:00 hrs. on 28/07/10 to 0800 hrs. on 29/07/10)		Mangla district of Punjab.
**28/07/10**	16:00	24:00	Heavy rainfall recorded in upper Khyber Pakhtunkhwa.Flash flooding expected in River Kabul and its tributaries during the next 24-36 hours.		Khyber Pakhtunkhwa.
**28/07/10**	19:00	00:30	Heavy rainfall expected in upper catchment of River Indus.River Indus at Tarbela region likely to attain Medium to High Flood levels (19:30 hrs on 28/07/10 to 10:00 hrs on 29/07/10).		Khyber Pakhtunkhwa
**28/07/10**	22:00	06:00	Heavy rainfall expected in upper catchment of River Indus. River Indus expected at Kalabagh likely to attain medium to high flood levels (04:00 hrs. on 29/07/10 to 22:00 hrs. on 29/07/10).		Kalabagh district of Punjab.
**29/07/10**	12:30	05:30	River Chenab at Trimu likely to attain medium flood level (18:00 on 31/07/10 to 08:00 on 02/07/10)		Expected riverine flooding in low lying areas of Districts Mandibahuddin, Chiniot, Jhang, Khanewal, Multan & Muzaffargarh districts of Punjab.
**29/07/10**	12:45	41:15	River Indus at Tanusa likely to attain high to very high flood level (06:00 on 31/07/10 to 06:00 on 01/08/10).		Expected riverine flooding in low lying areas of Districts Bhakkar, Layyah, Muzaffargarh, DG Khan and Rajanpur of Punjab
**29/07/10**	19:30	00:30	Heavy rainfall expected in upper catchment of River Jhelum.River Jhelum at Mangla likely to attain high to very high to exceptionally high flood level (20:00 on 29/07/10 to 08:00 on 30/07/10)		Mangla district of Punjab.
**30/07/10**	10:30	5:30	River Indus at Kalabagh likely to attain a very high to exceptionally high flood level (16:00 on 30/07/10 to 18:00 on 31/07/10)		Kalabagh district of Punjab.
**30/07/10**	10:40	17:20	River Indus at Chashma likely to attain a very high to exceptionally high flood level (04:00 on 31/07/10 to 20:00 on 31/07/10)		Mianwali district of Punjab.
**31/07/10**	13:40	29:20	River Indus at Taunsa likely to attain exceptionally high flood level (18:00 on 01/08/2010 to 18:00 on 03/08/10).		Expected riverine flooding in low lying areas of Districts Bhakkar, Layyah, Muzaffargarh, DG Khan and Rajanpur districts of Punjab.
**01/08/10**	10:15	~48:00	River Indus at Guddu likely to high flood level (on 03/08/10) and exceptionally high flood level (on 06/08/10). River Indus at Sukkur likely to attain high flood level (on 04/08/10) and exceptionally high flood level (on 07/08/10)		Riverine flooding expected in low-lying areas of Ghotki and Sukkur Districts in Sindh. Riverine flooding expected in low-lying areas of Sukkur, Larkana, Nawabshah, Hyderabad, Nosheroferoz Districts in Sindh.
**01/08/10**	15:40	1:20	Moderate flooding likely in Hill Torrents (17:00 on 01/08/10 to 08:00 on 02/08/10).		DG Khan and Rajanpur Districts of Punjab.
**02/08/10**	14:30	1:30	Moderate to heavy flooding likely in Hill Torrents (16:00 on 02/08/10 to 08:00 on 03/08/10).		DG Khan and Rajanpur Districts of Punjab.
**03/08/10**	10:00	00:30	Moderate to heavy flooding likely in Hill Torrents (10:30 on 03/08/10 to 23:00 on 03/08/10)		DG Khan and Rajanpur Districts of Punjab.
**03/08/10**	13:45	~36:00 ~10:15	River Indus at Guddu likely to exceptionally high flood level (on 05/08/10) and exceptionally high flood level (on 06/08/10). River Indus at Sukkur likely to attain high flood level (on 04/08/10) and exceptionally high flood level (on 07/08/10)		Riverine flooding expected in low-lying areas of Ghotki and Sukkur Districts in Sindh. Riverine flooding expected in low-lying areas of Sukkur, Larkana, Nawabshah, Hyderabad, Nosheroferoz Districts in Sindh.
**03/08/10**	21:00		Moderate to heavy rainfall in Khyber Pakhtunkhwa.Flash flooding is expected in River Kabul and its tributaries during next 24 hours.		Khyber Pakhtunkhwa
**03/08/10**	22:00	00:30	Moderate to heavy flooding likely in Hill Torrents (22:30 on 03/08/10 to 12:00 on 04/08/10)		DG Khan and Rajanpur Districts of Punjab.
**04/08/10**	11:30	00:30	Moderate to heavy flooding likely in Hill Torrents (12:00 on 04/08/10 to 24:00 on 04/08/10)		DG Khan and Rajanpur Districts of Punjab.
**05/08/10**	01:00	04:00	Moderate to heavy flooding likely in Hill Torrents due to rain in Suleman range (05:00 on 05/08/10 to 17:00 on 05/08/10)		DG Khan and Rajanpur Districts of Punjab.
**05/08/10**	11:00	24:00	RED ALERT: River Indus at Guddu likely to attain high to exceptionally high flood level (on 05/08/10 to 06/08/10). RED ALERT: River Indus at Sukkur likely to attain high flood level (on 06/08/10) and exceptionally high flood level (on 07/08/10 to 08/08/10)		Riverine flooding expected in low-lying areas of Khairpur, Jacobabad, Sadiqabad, Shikarpur, Ghotki, Sukkur in Sindh. Riverine flooding expected in low lying areas of Dadu Sukkur, Larkana, Nawabshah, Hyderabad, Nosheroferoz Districts in Sindh.
**06/08/10**	11.30	~06:00 156:30	River Indus at Sukkur likely to attain exceptionally high flood level (on evening of 06/08/10 to 07/08/10). River Indus at Kotri likely to attain a very high to exceptionally high flood-level (on 10/08/10 to 11/08/10).		Riverine flooding expected in low lying areas of Dadu Sukkur, Larkana, Nawabshah, Hyderabad, Nosheroferoz Districts in Sindh. Riverine flooding expected in low-lying areas of Thatta district and adjoining areas of the riverbed in Sindh.
**06/08/10**	14:00	01:00	River Chenab likely to attain high flood level (15:00 on 06/08/10 to 08:00 on 07/08/10).High flood is expected in River Chenab in Khanki area (22:00 on 06/08/10 to 13:00 on 07/08/10)		Low-lying areas of Gujranawala, Sialkot, Gujrat, Hafizabad, and Mandibahauddin districts on Punjab.
**06/08/10**	20:00	07:00	River Chenab likely to attain high flood level (03:00 on 07/08/10 to 15:00 on 07/08/10).	Low-lying areas of Hafizabad, Mandibahauddin, Chiniot and Jhang.	
**07/08/10**	11:30	00:00	Heavy rainfall has been recorded in the upper catchment of river Kabul its tributaries and more rainfall is expected.Flash flooding (high to Very High) is expected in River Kabul and its tributaries during the next 24-36 hours.Flash flooding is expected in Kurrum and Gambila rivers.	Low-lying areas of Sawat, Kalpani, Panjkora, Jindi, Adezai, Naugaman, Khiali, Shahalam, Chitral)	
**07/08/10**	16:00	01:00	Moderate to heavy flooding likely in Hill torrents due to heavy rain in Suleman range (17:00 on 07/08/10 to 05:00 on 08/08/110)	DG Khan and Rajanpur Districts of Punjab.	

## Appendix 2

### Cluster Groups involved in the Pakistan Floods 2010

**Table:**

**Cluster**	**Primary Governmental Counterpart**	**Cluster Lead Agency**
Agriculture	Ministry of Agriculture	FAO
Camp management/ Camp	National Disaster Management Authority and Provincial Disaster Management Authorities	UNHCR
Coordination	National Disaster Management Authority and Provincial Disaster Management Authorities	UNDP
Community Restoration	National Disaster Management Authority and Provincial Disaster Management Authorities	UNDP
Education	Ministry of Education	UNICEF/Save the Children
Food	National Disaster Management Authority and Provincial Disaster Management Authorities	WFP
Health	Ministry of Health	WHO
Logistics, Emergency Telecommunications	National Disaster Management Authority and Provincial Disaster Management Authorities	WFP
Nutrition	Ministry of Health	UNICEF
Protection	Ministry of Social Welfare	UNHCR
Shelter & NFIs	National Disaster Management Authority and Provincial Disaster Management Authorities	Ministry of Social Welfare
WASH	Ministry of Environment, Provincial Public Health Engineering Departments	UNICEF
